# Objective Structured Clinical Examination? How students perceive their learning after OSCE

**DOI:** 10.12669/pjms.37.4.4005

**Published:** 2021

**Authors:** Lamia Yusuf

**Affiliations:** Dr. Lamia Yusuf Associate Professor Gynae & Obs, Pak Red Crescent Medical College, Lahore, Pakistan

**Keywords:** OSCE, Learning, Clinical Assessments

## Abstract

**Objective:**

Assessment of skills is an important part of medical education. The objective was to find out how students perceive their learning after being assessed through OSCE.

**Methods:**

It was a mixed convergent parallel study. The study was carried out in the gynaecology department of a private medical college in Lahore. Five groups of final year students posted for rotation in gynae wards were included in this study. Each group comprised of 18 students. They were exposed to OSCE once daily. After completion of one month, students were given a feedback questionnaire (developed and validated by medical educationists) and a focus group discussion was carried out. Eight participants were selected from each group randomly and interviews were recorded. Interviews were transcribed verbatim.

**Results:**

Quantitative data calculated by SPSS 21 and results were calculated as frequencies. Qualitative analysis was based on word frequency count and thematic analysis was followed. Almost 65.2% of the student Shows agreement to the response that OSCE should be part of internal assessments. Whereas 15% shows disagreement and 9% remain neutral. The themes extracted after interviews were, better assessment tool, exam fear, knowledge, confidence and change learning style. thus, triangulating the data. The outcome measured was the result of the final professional of obstetrics and gynaecology year 2017-2018 batch. The success rate was 100% in gynae/obs and one student was able to get a distinction.

**Conclusion:**

Students perceived OSCE as a better assessment tool that increased their knowledge and confidence level. Thus, they were an opinion that OSCE should be part of examinations held in college.

## INTRODUCTION

Assessment drives learning as said by E miller. Assessment is developed according to objectives and tools of learning. During the past few years trend has been changed from the assessment of learning to assessment for learning. According to Miller’s hierarchical triangle of assessments, shows, and shows how are assessed by different tools but objectively structured clinical examination is one of them. In Pakistan, traditional teaching is being followed for years, thus focusing on knowledge only, and unstructured long cases are still used to assess clinical skills, but these tools are not reliable to measure skills and attitude of students. In last few years, OSCE has been introduced by some institution in the curriculum as an assessment tool, but still, it is not being used regularly by many institutions.

The OSCE is thought to be an effective tool to evaluate clinical competence and is considered more reliable, consistent and valid than traditional clinical assessment approaches. Due to its high validity and reliability, OSCE is considered to be a yardstick for clinical assessment.^1^ Professor Harden wrote in his book that OSCE is a circuit of stations that involves clinical scenario and tasks that each student should perform in a circuit.^2^ An OSCE was defined as a method to evaluate student’s performance of specific skills in simulation. Harden and Gleeson (1979) first introduced the idea of the Objective Structured Clinical Examination (OSCE). It requires that examinee should move in a circuit through different stations’ in which they were presented with a clinical scenario. They had to demonstrate specific clinical examination skills in a specific prefixed time.^1^,^2^ A main feature of the OSCE was that each student performed the same series of tests. The OSCE was one of the first performance-based exams that were used to assess physician competence and are now considered the epitome of performance-based assessment in medicine.^3^

As already discussed, OSCE is being used to assess behaviours under control condition, in other word Shows and Shows how level in the hierarchical triangle. So the areas that are most critical for performing skills of a doctor is being assessed through OSCE. These areas include communication skills, counselling skills, data interpretation, breaking bad news and skills demonstrated on patient or manikins. It is different from the traditional long case where usually one or two examiners assess limited skills. through OSCE, we were able to assess a different aspect of obs & gynae.^4^

OSCE is having a few shortcomings as well. First of all, the faculty must be trained to construct OSCE. Faculty should be well versed with Bloom’s taxonomy and Miller’s triangle of assessments. Great preparation time is required before the examination. Keys should be made according to the preset rubrics. Another disadvantage of this approach may be an understanding that the student’s knowledge and skills are channeled into compartments. Traditional long case redesigned in a structured manner can be used to evaluate this part of the assessment. Finally, patients must be selected carefully for the examination and the questions organized to cause the patient the minimum of disturbance. For this purpose, a group of simulated patients should be made.^5^

These shortcomings require an audit or evaluation of OSCE. Students’ perception can be a way to provide an opinion about implementing and improvement of this methodology. The objective of this study was to appreciate the perception of students about their learning after being assessed through OSCE, as they are the end-users and they are going to be benefited more.

## METHODS

The study was carried out in the gynaecology department of a private medical college in the year December 2017 - September 2018 (whole academic year). It was a mixed convergent parallel study. Permission from the ethical review board of institution was taken. Informed consent was taken from students. Five groups of final year students posted for rotation in gynae wards were included in this study. Each group comprised of 18 students. These groups join the gynae department after rotating through other wards All examiners and faculty members of the department were trained and briefed about how to make OSCE stations and how to conduct OSCE. Students were given themes, topics and symptoms to prepare for OSCE. The schedules were informed to them at the start of ward rotation. They were exposed to OSCE once daily. Each OSCE circuit consisted of 15 stations, five out of these were interactive or observed stations (according to the TOS of UHS). On these five stations students were asked to perform certain tasks, to check interpretation, analysis and implementation skills (knows, knows how, shows and shows how).

After each OSCE students went through post OSCE feedback sessions and in this session their shortcomings were discussed. After completion of one month, students were given a feedback questionnaire (developed and validated by medical educationists) and focus group discussions were carried out. Eight participants were selected from each group randomly and interviews were recorded. Interviews were transcribed verbatim. These interviews were conducted by a medical educationist with a sound background of research.

## RESULTS

Quantitative data were calculated as frequencies by SPSS 21 and for qualitative analysis, NVIVO was used to do thematic analysis and to create a word cloud. The themes derived after interviews were better assessment tool, exam fear, knowledge, confidence and change learning style.

## DISCUSSION

Objectively Structured Clinical Examination (OSCE) is a vital part of the assessment of clinical skills. It is a more structured and objective assessment of students’ clinical competence. This assessment tool examines a wide range of students’ knowledge, skills and competencies. This study employed a mixed methodology to determine the perception of students about their learning after OSCE. Both the result of quantitative and qualitative analysis endorsed each other.

Almost 65.2% of the student agreed to the response that OSCE should be part of internal assessment, whereas 15% disagreed and 9% remain neutral. These results were comparable to another study.^6^ The findings are established during in-depth interviews of the participant, each group endorsed that OSCE should be part of internal assessment. Students agreed in a similar study by Hammad et al.^7^

Sixty-six % of the students in the study has agreed that OSCE has been able to achieve the learning objective (Table-I). This fact was triangulated in the focus group discussion here students affirmed that OSCE has improved their knowledge (Table-II). The same agreement was found in another study.^8^

**Table-I:** Perception of Students about Learning after OSCE.

	Strongly disagree	Disagree	Neither agree/ Nor disagree	Agree	Strongly agree	No response
Does OSCE focus on learning objectives	2(2.9)		1(1.4)	36(52.2)	30(43.5)	
Does OSCE covers all the topics in Gynae/OBS	2(2.9)	9(13.0)	11(15.9)	34(49.3)	13(18.8)	
Does OSCE tested practical skills	6(8.7)	12(17.4)	16(23.2)	25(36.2)	10(14.5)	
Does OSCE help to identify your learning deficiencies?	3(4.3)	2(2.9)	10(14.5)	29(42.0)	25(36.2)	
Were you comfortable throughout the process?	2(2.9)	5(7.2)	15(21.7)	36(52.2)	10(14.5)	1(1.4)
Do you think you need more time on each station?	8(11.6)	34(49.3)	5(7.2)	16(23.2)	5(7.2)	1(1.4)
Was the OSCE were well organized	1(1.4)	8(11.6)	10(14.5)	34(49.3)	14(20.3)	2(2.9)
Was the exam stressful?	5(7.2)	21(30.4)	18(26.1)	14(20.3)	11(15.9)	
Is OSCE a fair examination as compared to the traditional exam?	1(1.4)	2(2.9)	8(11.6)	41(59.4)	17(24.6)	
Is it easy to score better in OSCE then traditional exam?	1(1.4)	7(10.1)	14(20.3)	32(46.4)	14(20.3)	1(1.4)
Does OSCE reduce the chances of bias ness by the examiner?	5(7.2)	8(11.6)	20(29.0)	25(36.2)	11(15.9)	
Should OSCE be included as an assessment tool in college examination?	4(5.8)	11(15.9)	9(13.0)	29(42.0)	16(23.2)

**Table-II:** Thematic Analysis.

Themes	Verbatim
The better assessment tool,	Better ability to understand the answers in clinical scenarios
Exam fear,	Familiarization of the procedure, feedback, repetition and practice are the reason that we will perform good/ well in final professional
Knowledge,	We have core knowledge we can interpret things well and perceive things and execute very well
Confidence	When you know things it automatically improves your confidence because now we know a lot about gynae so that improves the confidence
Change learning style	it completely changed your entire thinking and the way of learning Self-learning

OSCE is preferred over a traditional exam as OSCE gives a greater chance to score as compared to the traditional exam, this is believed by 46% of students in this study. The reason can be the nature of the OSCE exam as all students have fixed time and are exposed to similar stations.^9^

OSCE is a better assessment tool to assess clinical skills. Participants in our study verify these results as shown in Table-I & II. The famous notion assessment drives learning is fully revealed in our study. Students reflected that OSCE has changed their learning style. They started thinking critically and they have become a self-directed learner. There are different type of learning strategies, and studies have shown that learner adopts learning strategies according to the objectives of the assessment. The nature of OSCE promotes deep learning among students.^10^ OSCE endorses critical thinking among students thus empowering them to improve their clinical competencies.^11^

**Fig.1:**
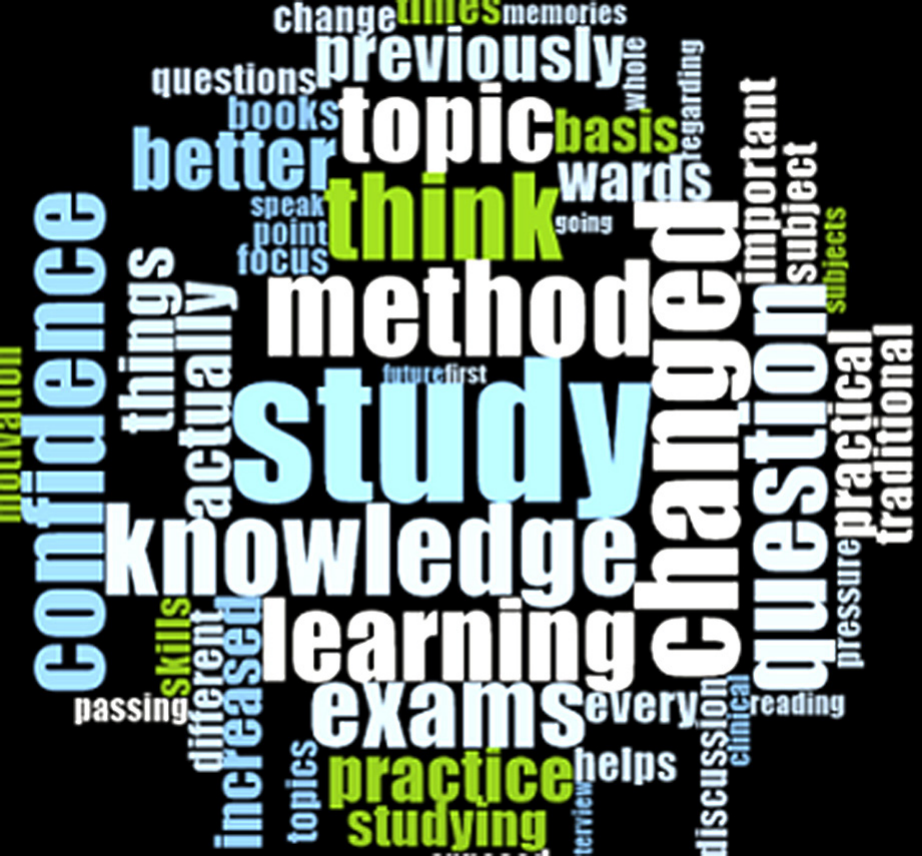
Word Cloud.

In our study, 20% of the coded items indicates that fear of examination has been reduced after OSCE. As shown in Table-II the reason for this is practice, familiarity with the procedure and feedback given after OSCE, all these promote deep learning.^12^ Fifty-four percent of participants in our study confirmed that OSCE discourses their learning deficiencies (Table-I), aids in focusing their learning objective and help in better performance. The nodes coded during the analysis of in-depth interview further affirmed these findings (Table-II). Similar findings were noted in another study.^13^

In this indexed study, 29% of the students believed that OSCE is quite stressed full but 27% disagree with this fact. These findings were similar to the finding described in the study carried out by Mamatha SD et al.^13^

Seven percent of students believed that it is easy to perform and score better in OSCE than the traditional examinations. 14 The reason is fairness in the structure of OSCE. All students are exposed to similar stations, and similar marking scheme, so that it removes the chances of prejudice.^4^,^9^,^11^

Though this data was collected before the COVID-19 pandemic, the importance of OSCE can’t be ignored in this global pandemic. Teaching & Learning has to be continued under strict SOPs in current social distancing requirements. In Pakistan institutions and universities are now switching to online OSCE to gain the full benefits of this assessment tool to assess clinical skills. Orientation programmes for students and faculty should be conducted to make them familiarize with synchronous online OSCE.^15^

### Limitations

The limitation of this study was the limited number of students. A large cohort of the students should be used to generalized the results.

## CONCLUSION

The study was able to determine the perception of the students. overall, the insight understanding of students regarding OSCE was very positive, they took it as a fair instrument of assessment with less chance of injustice, also they wanted to make it an integral part of internal assessment of college.
